# [Retracted] Effect of apelin on the cardiac hemodynamics in hypertensive rats with heart failure

**DOI:** 10.3892/ijmm.2025.5536

**Published:** 2025-04-22

**Authors:** Hui Pang, Bing Han, Tao Yu, Zhenkun Zong

Int J Mol Med 34: 756-764, 2014; DOI: 10.3892/ijmm.2014.1829

Following the publication of the above article, a concerned reader drew to the authors' attention that, regarding the western blot data shown in Fig. 7B on p. 762, a number of the bands shown to represent phosphorylated (p)-ERK-1/2 bands were strikingly similar to bands that were showing the results from the total (t)-ERK-1/2 experiments; moreover, certain of the bands in question were non-contiguous in terms of how they were positioned in the gels.

Upon examining these data independently in the Editorial Office, the Editor of *International Journal of Molecular Medicine* has determined that this article should be retracted from the Journal on the basis of an overall lack of confidence in the presented data. The authors were asked for an explanation to account for these concerns, but the Editorial Office did not receive a reply. The Editor apologizes to the readership of the Journal for any inconvenience caused.

